# Case Report: Renal metastasis of a gastric gastrointestinal stromal tumor mimicking a primary renal carcinoma

**DOI:** 10.3389/fonc.2025.1669412

**Published:** 2025-10-01

**Authors:** Xiaoxiao Bao, Chunyan Chen, Bifei Huang

**Affiliations:** Department of Pathology, Affiliated Dongyang Hospital of Wenzhou Medical University, Dongyang, Zhejiang, China

**Keywords:** gastrointestinal stromal tumor, distant metastasis, kidney, treatment, prognosis

## Abstract

**Introduction:**

Gastrointestinal stromal tumors (GISTs), mesenchymal neoplasms of the gastrointestinal tract, are prone to recurrence and metastasis. Despite improvements in prognosis with targeted treatments, such as imatinib, some patients develop metastatic GIST. Renal involvement in GIST is uncommon, and data on the diagnosis and treatment of GIST with renal metastasis remain limited. In this report, we describe the diagnostic and therapeutic regimen used for a patient with renal metastasis from GIST, providing an important reference for clinicians.

**Case presentation:**

A 52-year-old man underwent resection of a gastric GIST 5 years previously and had received adjuvant imatinib therapy for 2 years. A radical nephrectomy was performed, and histopathological analysis confirmed metastatic GIST. Although the patient resumed imatinib therapy post-surgery, follow-up imaging 22 months later revealed multiple metastases in the retroperitoneum and abdominal cavity.

**Conclusion:**

Renal metastasis from GIST is highly aggressive. Although surgical resection offers considerable palliation and may prolong survival, survival is generally dependent on sustained multimodal therapy and follow-up. Early detection and management of recurrent or metastatic lesions are imperative to optimize long-term outcomes.

## 1 Introduction

Gastrointestinal stromal tumors (GISTs) are mesenchymal tumors originating from the gastrointestinal tract (1, 2). The pathogenesis of GIST is complex, primarily involving mutations in the *KIT* or *PDGFRA* genes (3, 4). Despite a recent rise in the incidence of GIST, clinical diagnosis and management remain challenging. Whereas the liver is a common site of GIST metastasis, renal involvement tends to be rare. Kidney-metastatic GIST may mimic primary renal carcinomas, with small tumors typically being asymptomatic, whereas large tumors can cause flank or abdominal pain, hematuria, and abdominal masses.

In this report, we describe a retrospective analysis of the clinical and pathological features, treatment, and prognosis of a patient with kidney-metastatic GIST treated at Dongyang People’s Hospital, aimed at providing a reference for clinicians managing similar cases.

## 2 Case description

On October 21, 2020, a 52-year-old man presented with intermittent-to-severe pain in the left abdomen and flank, which had persisted for 6 months and had worsened over the preceding 12 days. The pain, although non-radiating, was severe. The patient denied having any associated symptoms, such as nausea, vomiting, hematemesis, melena, chest tightness, dyspnea, altered bowel or bladder habits, dysuria, hematuria, or constitutional weakness.

On physical examination, the abdomen was soft and non-distended. The left upper abdomen was full, and a firm mass measuring 15 × 12 cm, with poor mobility and adhesion to surrounding tissues, was palpated. Although the overlying skin showed no evidence of redness or swelling, mild tenderness without rebound pain was detected, and percussion over both renal angles elicited no discomfort.

**Figure 1:**
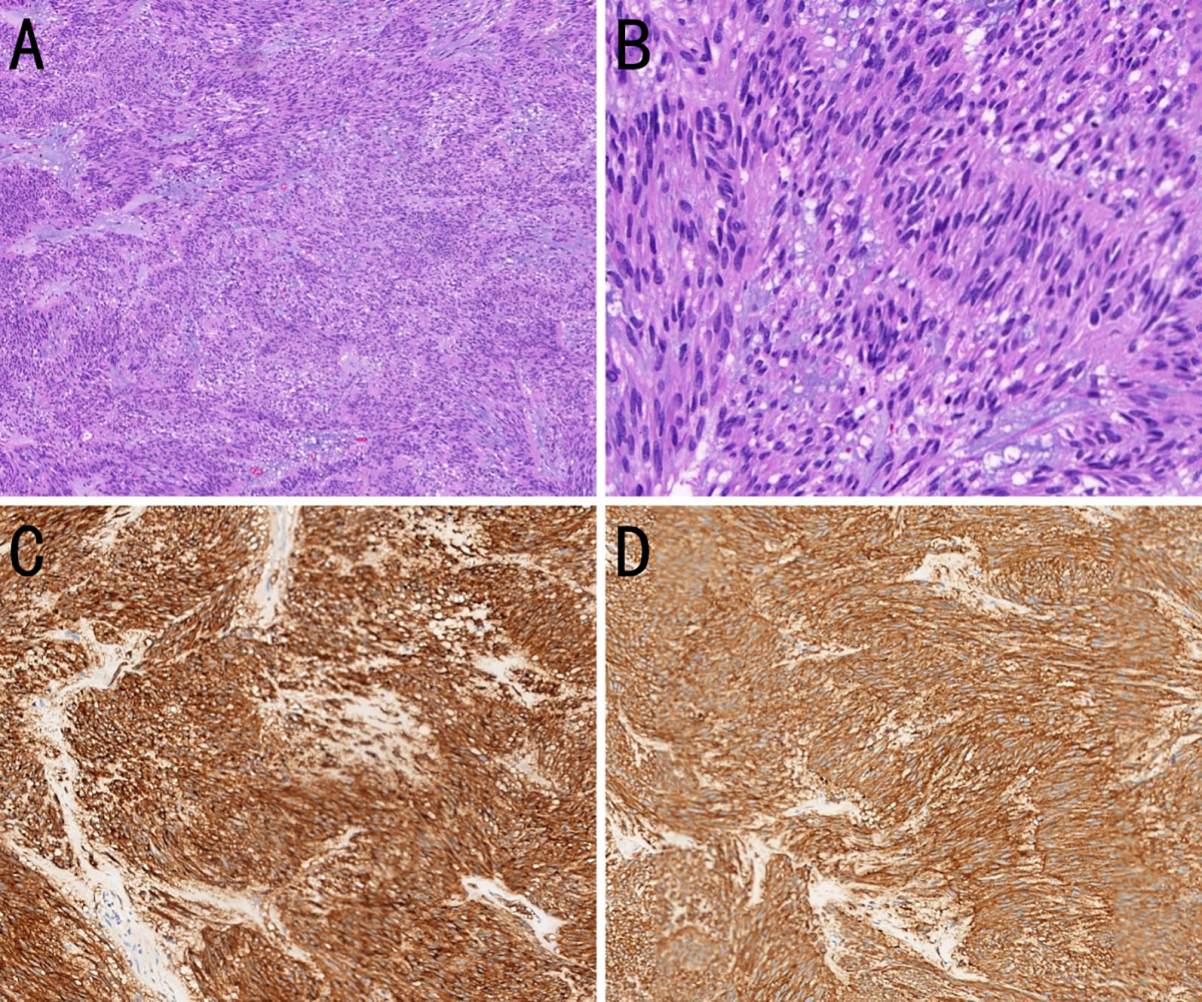
Histopathological and immunohistochemical features of a gastric gastrointestinal stromal tumor (GIST). **(A)** The tumor cells are arranged in bundles with myxoid stromal change. [hematoxylin and eosin (H&E), ×100]. **(B)** The tumor cells are spindle-shaped or short spindle-shaped (H&E, ×400). **(C)** The tumor cells strongly express CD117 (immunohistochemistry, ×200). **(D)** The tumor cells strongly express DOG-1 (immunohistochemistry, ×200).

Approximately 5 years prior to presentation (July 15, 2015), the patient had undergone laparotomy with a partial gastrectomy and splenectomy under general anesthesia for resection of a gastric tumor. The tumor, measuring 7 × 6 × 5 cm on the greater curvature near the cardia, had penetrated the serosa and extended to the splenic hilum and pancreatic tail, involving the splenic vessels. Several enlarged lymph nodes were palpable around the area. Postoperative pathology revealed an intermediate-risk GIST (**Figure 1**). The tumor, measuring 5.5 × 5.0 cm, was positive for smooth muscle actin, CD117, and CD34; and negative for S-100, with a Ki-67 proliferation index of 15%. The resection margins were negative, and he completed 2 years of oral imatinib (400 mg once daily) without evidence of recurrence during follow-up. The timeline, including key events, is summarized in **Table 1**.

**Table 1:** Chronology of the key diagnostic and therapeutic events from the initial gastrointestinal stromal tumor (GIST) resection (2015) to renal metastasis management and subsequent disease progression 22 months after nephrectomy.

Date/Interval	Event
July 15, 2015	Partial gastrectomy and splenectomy for a moderate-risk gastric GIST. Adjuvant imatinib was administered from 2015 to 2017.
October 21, 2020	Worsening left-sided abdominal and flank pain, leading to hospitalization.
October 24–27, 2020	Computed tomography and magnetic resonance imaging identify a solitary left renal mass.
November 2, 2020	Radical left nephrectomy performed; pathology confirmed metastatic GIST.
November 7, 2020	Postoperative imatinib therapy restarted at 400 mg once daily.
22 months post-nephrectomy	Follow-up imaging revealed multiple retroperitoneal and abdominal metastases, consistent with disease progression.

## 3 Diagnostic assessment

**Figure 2:**
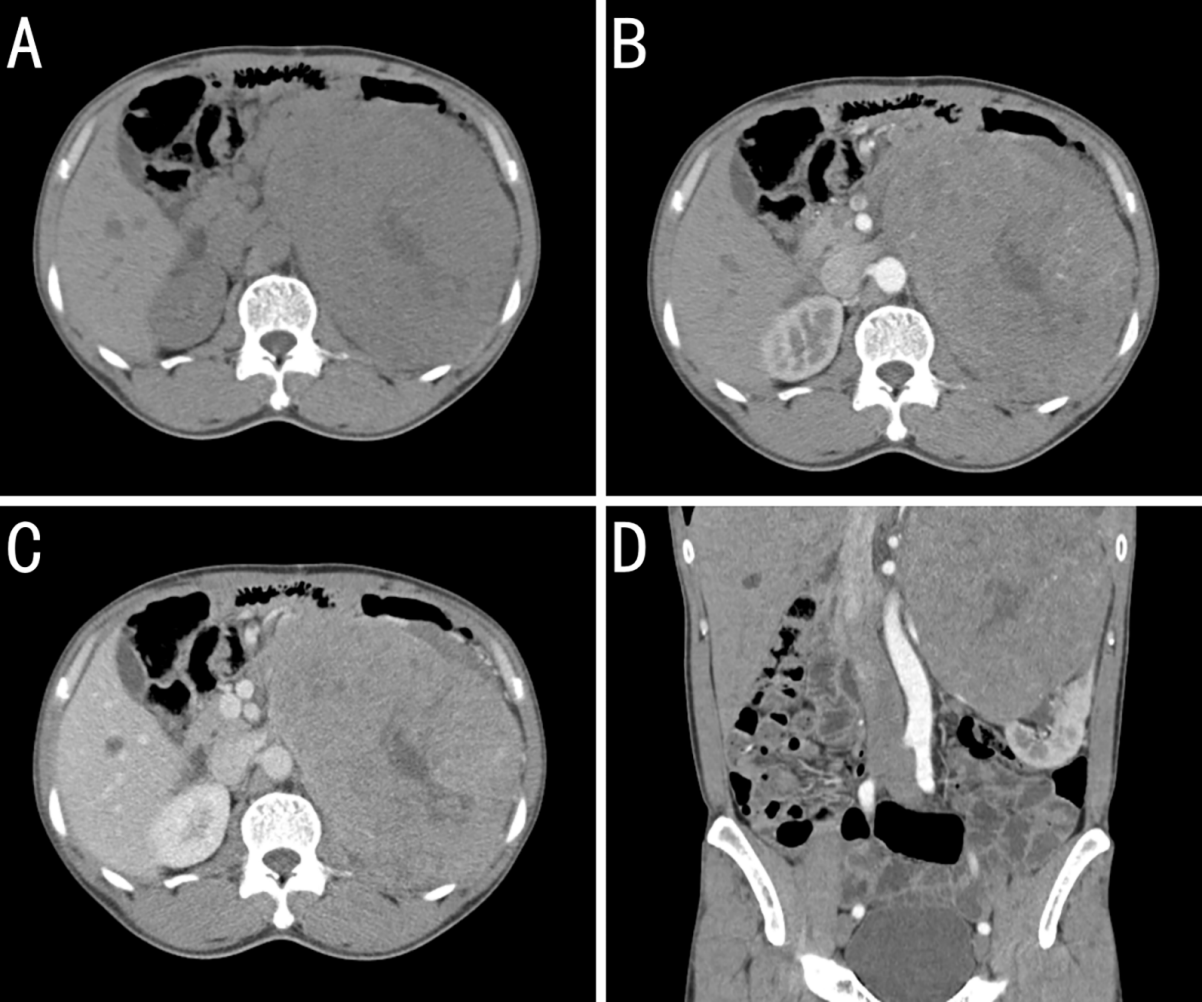
Contrast-enhanced computed tomography (CT) findings for a kidney-metastatic gastrointestinal stromal tumor (GIST). **(A)** Plains showing a large left retroperitoneal mass of near-equal attenuation containing patchy low-density foci. **(B)** Arterial phase image revealing mild heterogeneous enhancement with multiple tortuous arteries and intralesional arteries, whereas the low-density foci remain unenhanced. **(C)** Venous-phase image showing continued enhancement of the solid components, whereas the low-density areas seen on the plain scan remain unenhanced. **(D)** Coronal arterial phase reconstruction showing the arterial supply from branches of the left renal artery. The mass obscures the boundary with the upper pole of the left kidney, which is compressed and displaced inferiorly.

On October 24, 2020, retroperitoneal computed tomography (CT) revealed a left retroperitoneal mass measuring 16.3 × 12.0 cm with relatively well-defined margins abutting the left kidney. Heterogeneous low-density areas were present, and contrast enhancement was uneven. However, no enlarged regional lymph nodes were observed. Retroperitoneal CT revealed a large left-sided retroperitoneal mass, suspected to be of renal origin (renal carcinoma), and magnetic resonance imaging (MRI) with contrast was recommended (**Figure 2**).

On October 27, 2020, contrast-enhanced MRI confirmed a left renal mass measuring 15.7 × 13.6 cm with a slightly lower signal on T1-weighted images, a heterogeneously high signal on T2-weighted images, and fat-suppressed sequences showing restricted diffusion on diffusion-weighted images, with marked heterogeneous enhancement during the arterial phase and reduced enhancement in the parenchymal phase. Collectively, these findings provided strong evidence of malignancy.

## 4 Therapeutic intervention and pathology

**Figure 3:**
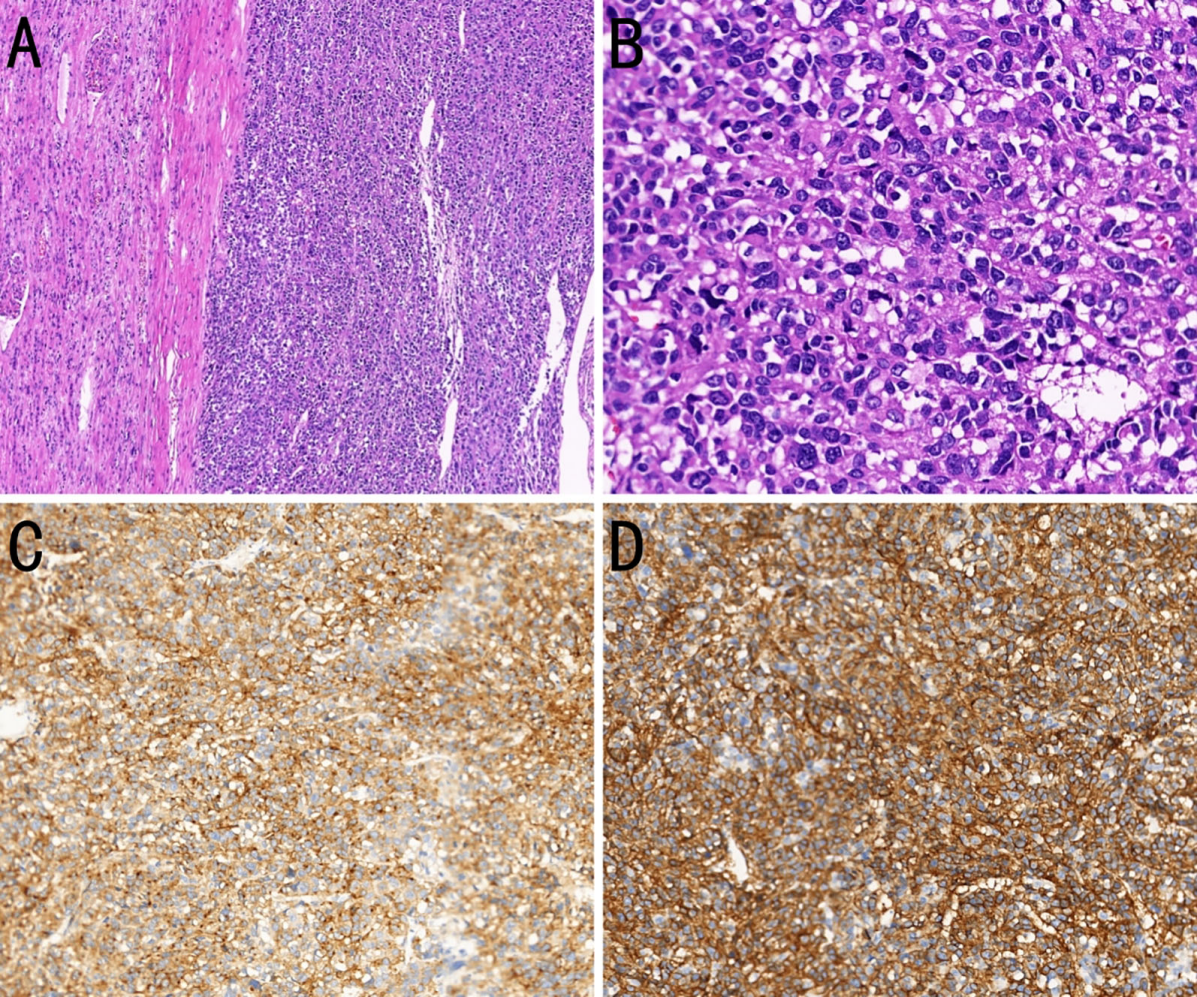
Histopathological and immunohistochemical features of a kidney-metastatic gastrointestinal stromal tumor (GIST). **(A)** Composite section showing normal renal parenchyma above and epithelioid-cell GIST below (H&E, ×100). **(B)** Magnified view showing epithelioid tumor cells with round to oval nuclei, clear to eosinophilic cytoplasm, frequent mitoses, and extensive necrosis (H&E, ×400). **(C)** The tumor cells strongly express CD117 (immunohistochemistry, ×200). **(D)** The tumor cells strongly express DOG-1 (immunohistochemistry, ×200).

On November 2, 2020, the patient underwent radical left nephrectomy, with a tumor measuring 18 × 11 × 10 cm being resected. Postoperative pathology revealed histology and immunohistochemistry consistent with a high-risk GIST (**Figure 3**). Subsequently, immunohistochemical analyses indicated positivity for CD117,CD34, discovered on GIST-1 (DOG-1) and vimentin; and negativity for smooth muscle actin, desmin, actin, S-100, and cytokeratin (CK). A Ki-67 proliferation index of approximately 70% was identified. The perinephric fat, ureteral margin, and vascular invasion were negative of tumor. In addition, a *KIT* exon 11 mutation was identified. Imatinib therapy (400 mg once daily) was resumed on postoperative day 5.

## 5 Follow-up and outcomes

Although the patient continued to receive imatinib over the course of the 22-month follow-up, subsequent imaging revealed multiple metastases in the retroperitoneum and abdominal cavity, indicating disease progression.

## 6 Discussion

Metastatic renal tumors originate from extrarenal malignancies. Although the kidneys are not a common target, most solid cancers can undergo renal metastasis (5). GIST is derived from the interstitial cells of Cajal, which serve as gastrointestinal pacemaker cells. These tumors most commonly metastasize to the liver, followed by the lungs and bones, whereas renal metastasis is rare (6). To the best of our knowledge, solitary renal metastasis from a gastric GIST has not previously been reported in the English-language literature. Clinical manifestations generally resemble those associated with primary renal carcinomas, with patients often initially being asymptomatic. As the tumor progresses, patients may experience persistent or intermittent flank or abdominal pain, visible hematuria, or a palpable lump (7). However, although renal lesions are rare at the initial GIST diagnosis, they are typically detected during the advanced stages characterized by metastatic spread.

Imaging plays a particularly important role in diagnosing kidney-metastatic GIST. However, although CT and MRI can accurately determine the location, size, and morphology of a lesion, the wide spectrum of primary renal neoplasms tends to be characterized by a broad radiological overlap, thereby rendering definitive identification difficult. Consequently, pathological examination remains the diagnostic gold standard because it can distinguish the three histological subtypes (spindle cells, epithelioid cells, and mixed types), while also revealing characteristic immunoreactivity to CD117 and DOG-1. Histological analyses also contribute to excluding the probability of similar tumors, such as primary renal carcinomas, which express CK and paired box gene 8 rather than CD117 or DOG-1, and angiomyolipomas, which generally comprise fat tissue, smooth muscle, and thick-walled blood vessels and tend to be positive for human melanoma black 45 and Melan-A but negative for CD117 and DOG-1.

Under conditions in which the extent of GIST metastasis is restricted, surgical resection remains the standard intervention, and complete surgical removal of lesions can significantly improve patient prognosis (8). However, multifocal metastatic GIST typically requires multi-organ resection, thereby necessitating a thorough abdominal examination. Nevertheless, if the metastatic proliferation is too extensive for curative surgery, resection should be avoided, as a prolonged recovery can delay systemic therapy with imatinib, which is currently the mainstay treatment for primary and metastatic GIST. Consequently, surgical complexity and overall tumor burden are considered key determinants in establishing the resectability of metastatic GIST (9). Although imatinib offers an appreciable advantage for patients with metastatic GIST who are either inoperable or at high surgical risk, secondary *KIT* mutations frequently lead to resistance to imatinib within 18–24 months (10). Among promising alternative approaches, ongoing molecular research has prompted trials examining the efficacy of inhibitors of the mammalian target-of-rapamycin and immune checkpoint molecules (11).

In the case reported herein, the patient underwent partial gastrectomy for a moderate-risk gastric GIST in 2015 and maintained 2-year disease-free survival on adjuvant imatinib. Five years later, a large renal mass was detected, with radical nephrectomy confirming metastatic GIST, and imatinib therapy was initiated. However, despite an initial response, multiple retroperitoneal and intraperitoneal metastases emerged 22 months after nephrectomy, indicating the development of imatinib resistance and a poor prognosis. When kidney metastasis is confirmed, surgery can contribute to a significant improvement in outcomes, with imaging serving as an indispensable diagnostic aid, nevertheless, histopathology remains the diagnostic gold standard.

In conclusion, renal metastases from GISTs are exceedingly uncommon, and there is currently no established standard of care. Preoperative imaging frequently misdiagnosis these lesions as primary renal cell carcinomas; therefore, accurate diagnosis relies on histopathology, immunohistochemistry, and molecular profiling. Although complete surgical resection of large renal masses can provide rapid relief from symptoms, adjuvant imatinib remains mandatory. For patients who exhibit resistance, multidisciplinary teams of practitioners should design personalized, multimodal treatment plans to extend survival and optimize the quality of life.

## Data Availability

The original contributions presented in the study are included in the article/supplementary material. Further inquiries can be directed to the corresponding author.

## References

[1] Akahoshi K Oya M Koga T Shiratsuchi Y. Current clinical management of gastrointestinal stromal tumor. World J Gastroenterol. (2018) 24:2806–17. doi: 10.3748/wjg.v24.i26.2806, PMID: 30018476 PMC6048423

[2] Keung EZ Raut CP. Management of gastrointestinal stromal tumors. Surg Clin North Am. (2017) 97:437–52. doi: 10.1016/j.suc.2016.12.001, PMID: 28325196

[3] Schaefer IM Mariño-Enríquez A Fletcher JA. What is new in gastrointestinal stromal tumor? Adv Anat Pathol. (2017) 24:259–67. doi: 10.1097/PAP.0000000000000158, PMID: 28632504 PMC5608028

[4] Venkataraman V George S Cote GM. Molecular advances in the treatment of advanced gastrointestinal stromal tumor. Oncologist. (2023) 28:671–81. doi: 10.1093/oncolo/oyad167, PMID: 37315115 PMC10400151

[5] Wu AJ Mehra R Hafez K Wolf JS Kunju LP. Metastases to the kidney: a clinicopathological study of 43 cases with an emphasis on deceptive features. Histopathology. (2015) 66:587–97. doi: 10.1111/his.12524, PMID: 25406592

[6] Schrage Y Hartgrink H Smith M Fiore M Rutkowski P Tzanis D. Surgical management of metastatic gastrointestinal stromal tumour. Eur J Surg Oncol. (2018) 44:1295–300. doi: 10.1016/j.ejso.2018.06.003, PMID: 30131102

[7] Saeed F Osunkoya AO. Secondary tumors of the kidney: a comprehensive clinicopathologic analysis. Adv Anat Pathol. (2022) 29:241–51. doi: 10.1097/PAP.0000000000000338, PMID: 35249993

[8] Yonkus JA Alva-Ruiz R Grotz TE. Surgical management of metastatic gastrointestinal stromal tumors. Curr Treat Options Oncol. (2021) 22:37. doi: 10.1007/s11864-021-00837-0, PMID: 33743084

[9] Fairweather M Cavnar MJ Li GZ Bertagnolli MM DeMatteo RP Raut CP. Prediction of morbidity following cytoreductive surgery for metastatic gastrointestinal stromal tumour in patients on tyrosine kinase inhibitor therapy. Br J Surg. (2018) 105:743–50. doi: 10.1002/bjs.10774, PMID: 29579329 PMC7938825

[10] Ang C Maki RG. Contemporary management of metastatic gastrointestinal stromal tumors: systemic and locoregional approaches. Oncol Ther. (2016) 4:1–16. doi: 10.1007/s40487-015-0014-7, PMID: 28261637 PMC5315077

[11] Vallilas C Sarantis P Kyriazoglou A Koustas E Theocharis S Papavassiliou AG. Gastrointestinal stromal tumors (GISTs): novel therapeutic strategies with immunotherapy and small molecules. Int J Mol Sci. (2021) 22:493. doi: 10.3390/ijms22020493, PMID: 33419029 PMC7825300

